# Sengstaken-Blakemore tube for non-variceal distal esophageal bleeding refractory to endoscopic treatment: a case report & review of the literature

**DOI:** 10.1093/gastro/gou023

**Published:** 2014-04-27

**Authors:** Yen-I Chen, Alastair P. Dorreen, Paul J. Warshawsky, Jonathan M. Wyse

**Affiliations:** ^1^Division of Gastroenterology, McGill University, Jewish General Hospital, Montreal, Canada, ^2^Internal Medicine, McGill University, Jewish General Hospital, Montreal, Canada and ^3^Critical Care Medicine, McGill University, Jewish General Hospital, Montreal, Canada

**Keywords:** Esophagus, therapeutics, endoscopy, gastrointestinal hemorrhage

## Abstract

Non-variceal upper-gastrointestinal bleeding (NVUGIB) refractory to therapeutic endoscopy is a challenging situation. The following details a novel use for the Sengstaken-Blakemore tube in a case of severe ulcerative esophagitis after failure of conventional medical and endoscopic treatment. A 77-year-old man with a history of peptic ulcer disease developed massive hematemesis during a hospital admission. Initial gastroscopy revealed an adherent blood clot occupying the distal esophagus, extending to the gastric cardia and proximal fundus. Epinephrine was injected into and surrounding the clot; however, following the endoscopy the patient was hemodynamically unstable, requiring aggressive resuscitation. Repeat gastroscopy, following saline lavage, revealed active bleeding within severely ulcerated esophageal mucosa, immediately proximal to the gastro-esophageal (GE) junction. Despite apparent hemostasis following injection of epinephrine and electrocautery, the patient displayed clinical signs of continued bleeding. Furthermore, surgical and radiological interventions were precluded by the patient's hemodynamic instability. In an attempt to tamponade blood supply to the GE junction, a Sengstaken-Blakemore tube was inserted and placed under tension. Successful hemostasis was subsequently achieved and the patient remained stable. This is the first case to describe use of a Sengstaken-Blakemore tube in severe ulcerative esophagitis refractory to standard endoscopic management.

## INTRODUCTION

The Sengstaken-Blakemore tube has been well described as a salvage therapy in the management of bleeding esophageal varices since its advent in the 1950s [1]. As a rescue therapy, balloon tamponade has been used to provide successful hemostasis in variceal bleeding in up to 80% of patients [2]; the use of this technique is, however, associated with a high rate of complications, including aspiration pneumonia, esophageal perforation, mucosal necrosis, and respiratory compromise secondary to external compression on the trachea [3, 4]. We present a case of uncontrolled, non-variceal upper gastro-intestinal bleeding (NVUGIB) treated with a Sengstaken-Blakemore tube after the failure of conventional medical and endoscopic treatment.

## CASE REPORT

A 77-year-old man with a history of peptic ulcer disease was admitted to the Internal Medicine ward for the treatment of septic arthritis. During the hospitalization, he developed hemodynamic instability following acute, massive hematemesis of approximately 750 mL of fresh blood and clot (nadir blood pressure 74/36 mmHg, pulse 110 bpm). Endotracheal intubation was performed along with resuscitation with volume and blood product; a continuous infusion of pantoprazole was initiated and the patient was transferred to the Intensive Care Unit for urgent gastroscopy.

Initial gastroscopy revealed an adherent blood clot occupying the distal esophagus, extending to the gastric cardia and proximal fundus. The clot could not be dislodged despite attempts with a water jet and wire snare, and neither the underlying lesion nor bleeding site could be identified. Five milliliters of a 1:10 000 solution of epinephrine was injected into and surrounding the clot. The remaining mucosa, as far as the third stage of the duodenum, was unremarkable except for pallor. Blood work revealed an initial drop in hemoglobin from 115 g/L to 86 g/L with a normal INR and platelet count. Four units of packed red blood cells (PRBC) were administered and an infusion of norepinephrine was required to maintain adequate mean arterial pressure.

Despite aggressive initial management, the patient required an additional four units of PRBCs, five units of platelets and five units of fresh frozen plasma, along with increasing doses of norepinephrine. Therefore, aggressive gastric lavage with over 3 liters of normal saline was performed, followed by immediate repeat gastroscopy. The actively bleeding site was now identified as arising within severely ulcerated esophageal mucosa just proximal to the gastro-esophageal (GE) junction; no esophageal or gastric varices were present. The area was injected with 10 mL of 1:10 000 epinephrine solution and treated with coaptive electrocoagulation, with apparent hemostasis. However, the patient did not demonstrate prolonged stability and recurrently bled, with the need for increasing hemodynamic support. Interventional radiology and general surgery were consulted and a multi-disciplinary discussion concluded that the patient was too unstable for transfer to the angiography suite and surgical morbidity and mortality was prohibitive. Given the location of the bleeding, a decision was made to immediately place a Sengstaken-Blakemore tube. Only the gastric balloon was inflated in an attempt to tamponade the GE junction. The balloon remained inflated for four hours and was then deflated to minimize mucosal ischemia, followed by a final three hours of inflation. During this time, the patient achieved and maintained hemodynamic stability with no transfusion requirements nor intravenous pressure support. There were no further clinical signs of active bleeding and the patient was transferred to a medical ward two days after the intervention with the Sengstaken-Blakemore tube and was discharged home four days later.

## DISCUSSION

Management of NVUGIB can be challenging despite aggressive medical therapy and therapeutic endoscopy. When standard endoscopy fails, a decision for angioembolization or surgery is usually made. Classically, the Sengstaken-Blakemore tube has been used as a rescue therapy in acute gastro-esophageal variceal bleeding [2], with its routine use limited by the high rate of complications [3, 4]. Case reports exist of balloon tamponade being used as a temporizing measure before surgical or endovascular repair of aorto-esophageal fistulas and in the treatment of Mallory-Weiss tears [3, 5]. The above case is the first to describe the use of the Sengstaken-Blakemore tube in NVUGIB due to severely ulcerated esophagitis. Effective hemostasis with balloon tamponade was achieved through mechanical compression of the underlying blood vessel due to its proximity to the GE junction, allowing for sufficient clot formation while an infusion of proton pump inhibitor maintained an elevated pH environment for improved platelet aggregation and decreased fibrinolysis [6]. This was achieved using only the gastric balloon, thereby decreasing the risk of serious complications associated with inflation of the esophageal balloon.

In NVUGIB that has failed to respond to endoscopic treatment, angiographic intervention or surgery are usually viable options [7]. The angiographic approach can be quite effective and is usually viewed as a secondary alternative after failed endoscopy, particularly in patients deemed unfit for surgery. It is nevertheless accompanied by significant re-bleeding rates of 10–20% and a mortality rate greater than 25% [7, 8]. Angioembolization is also associated with significant complications, such as bowel and hepatic infarction [8, 9]. Emergency surgery in cases of upper gastrointestinal hemorrhage is associated with a mortality rate greater than 20% [10], which is in part due to the selection of patients with severe, uncontrolled hemorrhage [11].

Our decision to use a Sengstaken-Blakemore tube was based on the specific location of the culprit lesion, failure of endoscopy, severe hemodynamic instability prohibiting transfer of the patient to the angiography suite and patient comorbidities that made surgery prohibitive. Interestingly, our patient had many high-risk characteristics for re-bleeding following embolization, such as longer time to angiography, greater than six units of PRBCs transfused, previous surgery, hypovolemic shock, and >2 comorbidities [8].

Although we illustrate the possibility of using a Sengstaken-Blakemore in a NVUGIB, we do not suggest its use other than in carefully selected cases where all options are discussed between gastroenterologists, interventional radiologists, critical care specialists and surgeons. This is the first case to describe use of a Sengstaken-Blakemore tube in severe ulcerative esophagitis refractory to standard medical management and therapeutic endoscopy.

**Conflict of interest:** none declared.
